# Decision-Making Capacity in a Transgender Patient With Schizophrenia and Concerns for a Life-Threatening Skin Infection

**DOI:** 10.7759/cureus.57123

**Published:** 2024-03-28

**Authors:** Amal Shafi, Benjamin K Woo, Davin Agustines

**Affiliations:** 1 Psychiatry, Western University of Health Sciences, Pomona, USA; 2 Psychiatry, Olive View-University of California Los Angeles Medical Center, Los Angeles, USA

**Keywords:** paranoid schizophrenia, bacterial skin infection, decision-making capacity, transgender care, schizophrenia

## Abstract

Assessing patient decision-making capacity while adhering to the requests of patients with mental illness remains a great ethical challenge. In patients with severe mental illness, the assessment of decision-making capacity can be difficult, particularly when a care team is also trying to navigate cultural, educational, and linguistic barriers. It becomes especially complex in situations where the patient is not only diagnosed with a severe mental illness but also suffers from a comorbid medical illness that the patient refuses to have treated appropriately. Balancing patient wishes while respecting patient autonomy creates further issues when assessing decision-making capacity. As such, the following case presents a transgender man who suffers from schizophrenia with a persistent skin infection on the patient’s torso secondary to wearing a brassiere for an extended period. This case report addresses the intricacies surrounding patient decision-making capacity, specifically in the psychiatric population.

## Introduction

Schizophrenia is a severe mental illness with a disease burden that is a leading cause of disability worldwide [1,2]. Described as a disorder with symptoms of psychosis and disorganized thinking, schizophrenia has become an important point of consideration when determining patient's abilities to make appropriate decisions in their health management and treatment [3]. This sheds light on the ethical questions behind respecting and maintaining patient autonomy in clinical psychiatric practice. 

Patient autonomy is the founding principle regarding patient healthcare [4]. This basic principle gives patients the right to make their own medical decisions, even when their decision goes against medical advice [5]. A caveat to this principle, however, is to recognize when an individual patient is limited in the capacity to make appropriate decisions about their health. As such, the capacity to make decisions is a fundamental principle in health care and has been extensively studied [4]. Decision-making capacity is assessed on four fundamental aspects: the ability to comprehend information, appreciate the significance of the information disclosed, reason through risks and benefits, and express a choice regarding their healthcare [6,7]. When working with psychiatric patients who suffer from delusions and disorganized thought processes, it becomes essential to determine the patient's ability to make appropriate decisions. 

In cases where patients may have diminished capacity, it becomes essential to conduct thorough and timely evaluations to ensure that the most appropriate decisions are made for patient treatment [6]. We present a case of a transgender male patient diagnosed with schizophrenia who had developed severe skin infections but refused to undergo examinations due to schizophrenic symptoms of paranoia. A PubMed search identified only one similar case of marginal decision-making capacity in a schizophrenic patient with a leg infection who unfortunately passed after refusing hospital-recommended amputation [8]. 

In caring for this patient, ethical questions surrounding the balance between respecting patient autonomy and addressing mental health become the focus of his care. 

## Case presentation

The patient is a 45-year-old black with schizophrenia who is a transgender male (assigned female sex at birth) with a history of methamphetamine abuse. Intermittently homeless, he had not been compliant with the antipsychotic treatment regimen of risperidone that had repeatedly been prescribed. This patient self-presented to the emergency department six times over the past nine months, complaining of wounds on his torso. However, on each visit, he would either let a select few staff members examine him or completely deny visualization of the affected areas. Approximately two months before this episode, the patient again came to the emergency department with the same complaints, but due to his paranoia, he refused a physical examination as well as the removal of his brassiere. ED staff suspected that the brassiere was likely causing his wound and prescribed a course of oral sulfamethoxazole-trimethoprim and consulted with the Unrepresented Patient Committee due to concerns for worsened ulceration and infection of the wound. This committee consists of physicians, nurses, social workers, and hospital risk managers who work together to make decisions on behalf of patients who do not have a legally authorized surrogate [9]. The Unrepresented Patient Committee decided that the patient could engage in informed refusal at that time, and no further recommendations were given. The patient was discharged by himself after completing the oral antibiotic course.

**Figure 1:**
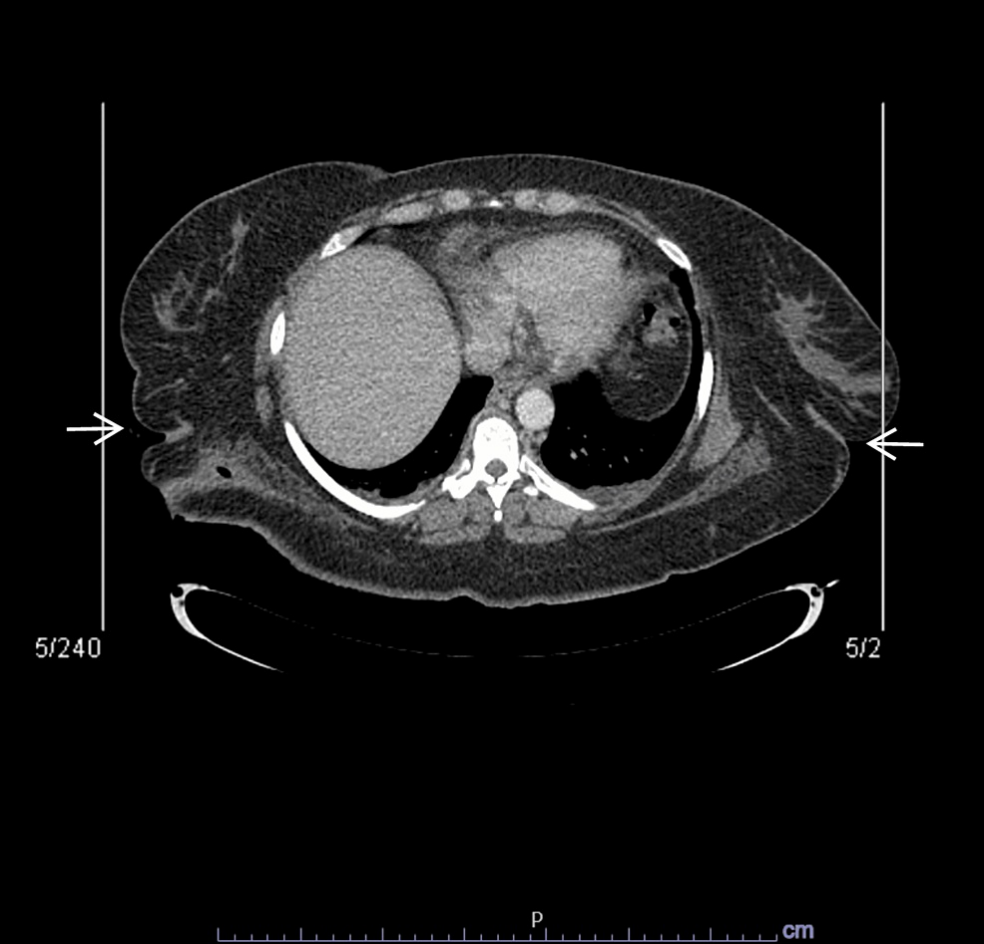
CT scan of the abdomen and pelvis

Four months after this last hospital visit, the patient self-presented once again to the emergency department with new-onset bleeding from his back and chest under the same brassiere. Despite his concerns, the patient again refused a physical examination. He was malodorous and paranoid and demonstrated evidence of disorganized thought processing. When questioned about his refusal of a physical examination, the patient would respond in fragmented sentences that lacked sense and had no relevance to the questions asked. Despite the patient’s then-stable vital signs, there was concern for a life-threatening infection, and the emergency department physicians concluded the patient could not refuse a physical examination. The emergency department physicians performed a two-physician override of the patient’s refusal to allow a physical exam. This process can be conducted in emergencies when concerns of life-threatening harm are present and consists of two independent physicians providing consent after independently reviewing the case at hand to agree on a medical plan as the best decision for the patient [10]. Once his undergarments were cut away, the patient was found to have severe skin infections across his chest, with bone exposed in several areas. He was then admitted to the inpatient medicine service for intravenous antibiotics as well as an evaluation for surgical debridement. They ordered a CT scan (Figure 1), which revealed some inflammatory changes noted in the subcutaneous tissue of the posterior chest wall with no discrete fluid collection. The surgical team concluded that no surgical intervention was necessary and recommended a more conservative course of wound care instead. The patient’s wounds were flushed with Vashe Cleaning Solution and covered with gauze, with instructions to change the dressing every three days. After finishing the intravenous treatment of ceftriaxone, piperacillin-tazobactam, and vancomycin, the patient was transferred to inpatient psychiatry to address the symptoms of paranoia and auditory hallucinations believed to be interfering with his care.

After the patient was admitted to the inpatient psychiatry unit, he downplayed the necessity for wound and medical care, continuing to make irrational statements about his treatment team, such as proclaiming treating staff to be chaplains.  He would not allow any staff to inspect the now-treated wounds or engage in a wound care regimen of wet-to-dry dressing changes with Vashe-soaked gauze. 

Though he complained daily of discomfort, the patient continued to refuse staff attempts to conduct dressing changes and wound examinations. Despite multiple attempts to engage the patient in a discussion about the risks and benefits of allowing dressing changes, he would always respond with, “No, thank you.” By day six of his inpatient psychiatric hospitalization, the patient had become noticeably malodorous, causing complaints from other patients and staff from across the unit. The patient also became progressively more agitated and yelled at anyone who attempted to come near him, demanding to be left alone. At one point, the patient even physically charged one of the physicians who asked to allow a physical exam. Because of the deteriorating clinical picture as well as the refusal to allow staff to inspect his wounds, another Unrepresented Patient Committee meeting was convened. This time, the committee approved the re-examination of his wounds, given his inability to provide any rationale for his refusal to allow care.

Once he was informed of the committee's decision, the patient decided to undergo the recommended laboratory and radiological tests. The results revealed a stable CT scan of the abdomen and pelvis and laboratory values that showed no signs of fever or leukocytosis. The inpatient psychiatry team arranged to have both infectious disease and medicine consult teams evaluate the patient simultaneously to decrease the potential amount of emotional trauma to him from multiple separate evaluations. Once the dressing was removed, the bilateral chest wounds were noted to be without purulence or drainage and appeared to be healing well. It turned out that the smell emanated from the bandages, which had begun to rot after he had worn them for 20 days straight without allowing a change. Once his wounds had sufficiently healed to allow the use of a brassiere, the patient was discharged to self. 

## Discussion

We find this case to be complicated from multiple standpoints. First, the patient experienced intermittent homelessness, complicating medication management and the follow-up of his mental illness. Additionally, he struggles with methamphetamine misuse and has been in and out of psychiatric hospitals for years. To add to his struggles, the patient is a transgender man who has tried to protect himself and feel safe in his homeless community by wearing a brassiere that was too small for his body. Due to a combination of fears about potentially losing his brassiere and chronic paranoia about being watched and followed, the patient refrained from removing his undergarment for months at a time. As a result, significant wounds developed on his chest and back.

Despite seeking medical care for his pain, the patient intermittently refused physical examinations secondary to his schizophrenia-induced paranoia surrounding healthcare workers. When approached to examine his wounds, his vague answers gave the illusion of assumed decision-making ability in the medical emergency room setting, which resulted in weeks passing without the patient getting appropriate medical care. His paranoia and concerns about being examined contributed to the recurring course of the patient’s illness, though his typical responses of “I just don’t feel comfortable with anyone looking at it” made it difficult for medical personnel to ascertain his immediate ability to engage in medical decision-making. Although the challenges of determining capacity are not unique to this patient population, the patient’s presentation of not only schizophrenia and limited decision-making capacity but also having a potentially life-threatening skin infection is rare, with only one other identified PubMed case presentation.

This case demonstrates the importance of evaluating decision-making capacity in patients dealing with both medical and psychiatric conditions. The goal is to create a balance between respecting patient autonomy and ensuring that appropriate decisions are made to avoid harm [6,11]. Although existing data validates the importance of determining decision-making capacity, the absence of capacity is not immediately apparent, and extra attention should be provided to ensure the best patient care [6]. In the case of the patient, the primary decision to respect his autonomy took precedence, but as concerns for infection began to rise, it became increasingly important to address his potential lack of capacity. While it was determined that the patient could not make appropriate decisions for himself, the medical team worked to bring the most comfort to the patient during this time. Additionally, recognizing the patient's concerns regarding his transgender identity, the care team arranged a designated appointment for physicians specializing in psychiatry, medicine, and infectious disease to collectively assess the patient's wounds. This approach aimed to minimize his discomfort, limiting the number of times the patient would have to undress for examination. Not only does this case emphasize the importance of consistently assessing patients’ ability to make sound decisions during their hospitalization, but it also highlights strategies aimed at promoting empathy within the transgender community.

## Conclusions

The ability to determine decision-making capacity can be challenging to assess and has become an important aspect to address when working with the psychiatric patient population. Paranoia, along with disorganized thought processes secondary to schizophrenia, can impact appropriate decision-making. As such, it becomes of utmost importance to continuously assess the four pillars of decision-making status intermittently throughout patients' hospital stays. During daily patient evaluations, it is important to encourage patients to articulate their health conditions and share their grasp of the risks and benefits of their plan of care to better assess their capacity. This case report and others of a similar nature highlight the ethical complexities of balancing patient autonomy in the psychiatric setting, all while in the face of potential illness.
